# Strengthening Leadership Pathways for Women Nurses and Midwives: Examining System and Organizational Opportunities in a Mixed‐Methods Study

**DOI:** 10.1155/jonm/9904118

**Published:** 2026-10-06

**Authors:** Mihirika Surangi De Silva Pincha Baduge, Helena J. Teede, Leanne Boyd, Ifeoluwa Adesina, Kathryn Joseph, Belinda Garth

**Affiliations:** ^1^ Monash Centre for Health, Research and Implementation Monash University Melbourne Victoria Australia monash.ac.za; ^2^ Institute of Health and Wellbeing Federation University Melbourne Victoria Australia federation.edu.au; ^3^ Intensive Care Eastern Health Melbourne Victoria Australia easternhealth.org.au; ^4^ Australian College of Nursing Canberra Australian Capital Territory Australia; ^5^ Endocrine and Diabetes Units Monash Health Melbourne Victoria Australia monashhealth.org; ^6^ Department of Health Systems and Equity Eastern Health Clinical School Monash University Melbourne Victoria Australia monash.ac.za

**Keywords:** gender equity, healthcare, leadership, midwives, nurses, women

## Abstract

**Background:**

The nursing and midwifery profession constitutes a substantial portion of the global health workforce, with the majority being women. Despite this, women nurses and midwives are underrepresented in healthcare leadership.

**Objectives:**

To explore the perspectives and experiences of nurses and midwives to identify system‐ and organizational‐level strategic opportunities for advancing women into leadership.

**Design:**

Two‐phased mixed‐methods design employing an exploratory sequential approach and weaving narrative methods.

**Setting:**

Large public and private healthcare services in Australia.

**Participants:**

Phase one included twenty‐five nursing and midwifery leaders. Phase two included 158 nurses and midwives.

**Methods:**

Data from the organizational change management stream of the Advancing Women in Healthcare Leadership national initiative was used. Phase one consisted of qualitative semistructured interviews and informed phase two, which comprised an online survey with qualitative and quantitative components. Qualitative data were analyzed thematically; quantitative data were analyzed descriptively.

**Results:**

System‐level opportunities for advancing women nurses and midwives into healthcare leadership included reforming legal agreements and challenging perceived hierarchies within professional roles. Organizational‐level opportunities included building leadership capacity through inclusive organizational policies and processes; fostering inclusive organizational cultures through awareness and engagement; expanding mentoring and networking opportunities; enhancing leadership development through accessible, tailored training; and embedding equity and transparency in recruitment and promotion.

**Conclusion:**

Advancing women nurses and midwives into healthcare leadership needs a shift from individual efforts to systemic strategies. Addressing structural barriers and fostering empowerment, healthcare systems can build inclusive, resilient leadership pipelines that reflect the diversity and expertise of the nursing and midwifery workforce.

**Patient or Public Contribution:**

This study examines nursing careers and workforce issues rather than nursing care. Consequently, no patients or members of the public were involved in its design, conduct, or reporting.

**Summary**
• What is already known
◦ Women nurses and midwives face significant challenges when pursuing leadership roles in health care, compounded by systemic and organizational structural barriers and gender biases.
◦ Strategies for advancing women into healthcare leadership often focus on individual development rather than addressing systemic and organizational barriers.
• What this paper adds
◦ System‐level reforms, such as changes to legal and policy frameworks and professional hierarchies, can remove structural barriers and create equitable pathways for women nurses and midwives to advance into healthcare leadership.
◦ Organizational strategies, including inclusive policies and practices, supportive workplace cultures, mentoring and networking opportunities, targeted leadership development, and transparent recruitment and promotion processes, are critical to enabling women nurses’ and midwives’ leadership progression.
◦ A shift from individual‐focused interventions to systemic and organizational approaches is essential to achieving sustainable gender equity in healthcare leadership for nurses and midwives.

## 1. Background

Nursing constitutes the largest proportion of the healthcare workforce, with 29 million nurses and 2.2 million midwives, accounting for 50% of all healthcare professionals globally [1, 2]. Despite women representing the majority of the nursing and midwifery professions, they remain underrepresented in healthcare leadership. As stated by the World Health Organization [3], healthcare is “delivered by women and led by men.” Women nurses and midwives encounter substantial challenges when aspiring to leadership, influenced by the intersection of gender and professional identity [4]. Cultural and societal stereotypes that frame nursing as a caregiving rather than a leadership profession [5], alongside gendered perceptions that associate women more closely with caring roles than with leadership, continue to significantly contribute to this issue [4].

Gender and the roles associated with it are shaped by social and cultural norms and evolve over time. While informed by the biological categorization of male and female, gender encompasses social and cultural differences in identity, expression, and experience as a man, woman, or nonbinary person [6, 7] and can influence access to societal roles and opportunities [8, 9]. In this study, we use the term “woman/women” inclusively to encompass all individuals who identify with this gender [7, 10]. This terminology acknowledges gender diversity while reflecting the specific focus of the study on barriers and opportunities associated with advancing women nurses and midwives into leadership positions.

Advancing the nursing profession leads to significant improvements in healthcare delivery, economic growth, peace, and overall societal well‐being [11]. Enhancing pay, working conditions, and career advancement opportunities in nursing empowers women by providing them with quality jobs and lifting them out of poverty [11]. This also helps to narrow the persistent gender pay gap that hinders global economic progress. Maximizing the contribution of nurses and midwives through leadership, policy engagement, and workforce capacity‐building is essential to achieving the Sustainable Development Goals of “leaving no one behind,” improving health for all, and building the primary healthcare workforce toward universal health coverage [12].

Despite the critical contributions of nurses and midwives to healthcare delivery, the underrepresentation of women nurses and midwives in leadership positions remains a significant issue. This disparity is compounded by a complex interplay of systemic barriers and gender biases, including organizational structures, limited access to opportunities, gender stereotyping, perceptions of professionalism, and inequitable human relations policies. These factors collectively influence motivation and access to leadership pathways. In this study, career pathways refer to the formal and informal routes through which nurses and midwives progress into leadership roles. These may include acting opportunities, secondments, mentoring, leadership programs, and promotion processes.

Additionally, the lack of visible role models and leadership development programs further constrains the ability of women nurses and midwives to advance into senior leadership positions [4]. Organizational cultures and policies often reinforce a “glass ceiling”—limiting upward mobility—and a “sticky floor”—confining women to lower‐paid, less influential roles and hindering their rise to higher leadership positions [14, 15]. These barriers are even more pronounced for women nurses and midwives from ethnically minoritized backgrounds, who face compounded challenges in accessing leadership opportunities [16].

Strategies for advancing women into healthcare leadership often focus on individual development (i.e., “fixing women”) rather than addressing systemic and organizational barriers [17–19]. Women must navigate existing structural obstacles that hinder their capacity, undermine their perceived capability, and diminish their credibility [17, 19]. Consequently, they often strive to succeed in a system ill‐equipped to support their career progression [20]. Further, most research centers on women in medicine, leaving a gap in understanding the unique solutions for women nurses and midwives to advance to healthcare leadership.

Knowledge of effective systemic and organizational strategies to advance women nurses and midwives into healthcare leadership remains limited. Therefore, this study aims to explore the perspectives and experiences of nurses and midwives to identify system‐level and organizational‐level strategic opportunities for advancing into leadership. System‐level opportunities refer to broader policy, regulatory, industrial, and societal influences, while organizational‐level opportunities relate to workplace policies, practices, culture, and leadership structures within healthcare organizations.

## 2. Methods

### 2.1. Data Source

This substudy focused on the advancement of women nurses and midwives in the context of the overarching study. A mixed‐methods design was employed, with an exploratory sequential approach [21]. Phase one involved qualitative interviews with healthcare leaders, which informed phase two, where an online employee survey was developed.

#### 2.1.1. Phase 1: Individual Qualitative Interviews With Leaders

Individual qualitative interviews (*n* = 113) were conducted to understand organizational contexts and what works at an organizational level from the perspective of leaders. Purposive sampling was used to identify women and men in leadership positions across disciplines including nursing, career stages, public and private sectors, culturally diverse groups, health service managers/leaders, and policymakers. Each partner nominated a senior leader/executive to email an Expression of Interest invitation to all managers, including Nurse Unit Managers and senior‐level nurse leaders. A participant information sheet was attached to the Expression of Interest. Interested leaders provided their contact details in the Expression of Interest, through a survey link developed by the research team. Screening questions were included in the Expression of Interest to determine whether the prospective participants met sampling requirements. Participation was voluntary and confidential, and health services did not know who responded to the Expression of Interest. Interviews were scheduled with those consenting to participate. All participants were emailed a participant information sheet and a summary of the interview questions in advance. Participants completed a brief demographic questionnaire before their interview to capture information across gender, age, cultural background, qualifications, employment arrangement, organizational affiliation, healthcare specialty, current leadership stage, location of work (e.g., metropolitan and regional), and unpaid care experience. Consent was obtained verbally by the interviewer immediately before each interview. Interviews were conducted online by experienced qualitative interviewers, between August 2021 and June 2022, utilized a semistructured interview guide framed by outcomes of evidence synthesis and the Consolidated Framework for Implementation Research (CFIR) [22, 23], were audio‐recorded, and lasted 45–60 min. Data were transcribed verbatim by an independent professional transcription service and deidentified. Qualitative software Dedoose was used to manage the data [24].

#### 2.1.2. Phase 2: Online Employee Survey

An online survey of healthcare employees, including nurses, midwives and other disciplines, and hospital administration, was conducted to understand organizational contexts and what works at an organizational level from the perspective of employees. All partner employees were invited to complete the online survey via email, newsletter advertisements, and social media channels from October 2023 to February 4, 2025. The survey was created and distributed using Qualtrics software [25].

#### 2.1.3. Data for This Study

To address the aim of this substudy, we used the data from nurses and midwives from the overarching dataset, from both Phase 1 and Phase 2. The data subset for this study included interviews from nursing and midwifery leaders and surveys from nurses and midwives. Figure 1 illustrates the data collection and analysis process.

**FIGURE 1:**
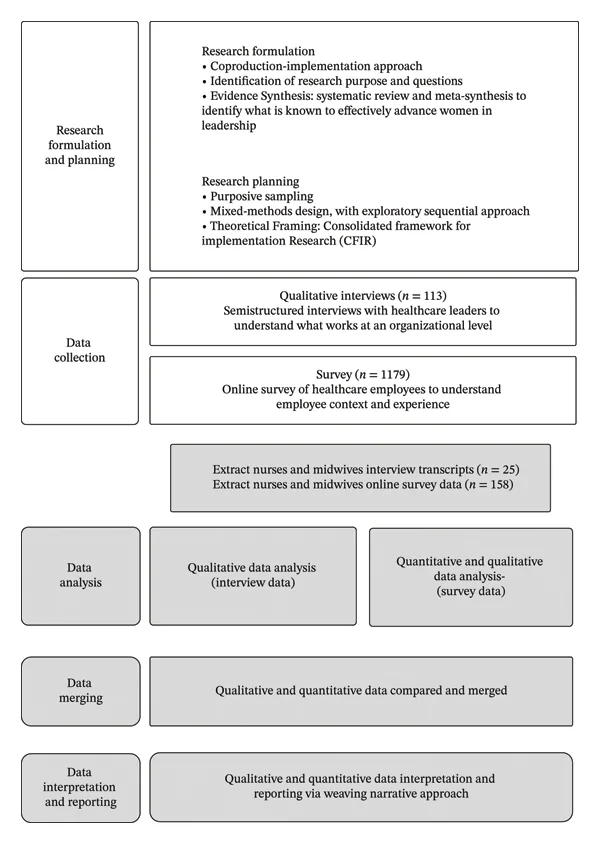
Data collection and analysis process.

### 2.2. Context

This study was nested within the Advancing Women in Healthcare Leadership (AWHL) national initiative, funded through two National Health and Medical Research Council Partnership Project Grants (APP1191837 and APP2018718) and partner contributions (partner contributions refer to financial and in‐kind support provided by AWHL partners. In‐kind support includes nonmonetary contributions, such as personnel time, professional expertise, facilities, equipment, and other resources) [26, 27]. The AWHL initiative employs coproduction‐implementation research methods, where stakeholders are engaged from the beginning with shared power and equal partnership. This approach integrates diverse perspectives, including local contexts, to influence the design, implementation, and delivery of interventions, to optimize their impact on policy and practice [27–30]. This study utilized data collected for the organizational change management stream of the AWHL national initiative. The original dataset consisted of interviews and surveys conducted with multidisciplinary healthcare professionals. For this analysis, interviews with healthcare leaders from nursing and midwifery backgrounds were combined with survey data from nurses and midwives, analyzed and triangulated to gain insights into employee‐level experiences.

Aligned with a coproduction approach, Advancing Women in Healthcare Leadership’s lead partner organizations were involved from the outset [27] and included two large public health services and two private health services in Australia. Partners worked with the AWHL team to develop objectives, data collection tools, and participant recruitment strategies. This active input continued throughout data collection, analysis, and dissemination to ensure shared findings and iterative improvements.

The aim of the overarching study was to identify systems and organizational strategies and priorities for achieving gender equality in healthcare leadership. Participants included leadership and workforce, with both required in transformational change. Gender was defined by self‐report, with participants selecting the identity that best reflected their lived experience. The study did not collect detailed information regarding transgender status, gender diversity, or sex assigned at birth. Consequently, analyses were based on participants’ self‐identified gender and experiences.

The overarching study was framed by the updated CFIR [23]. This provides a comprehensive structure to systematically assess and guide the implementation of health services research into practice, encompassing five major domains: outer setting, inner setting, characteristics of individuals, intervention characteristics, and the implementation process. CFIR helps to identify potential barriers and facilitators to effective implementation of evidence‐based interventions across diverse contexts. This framework emphasizes that alignment of all elements is needed to optimize career advancement.

### 2.3. Data Analysis

#### 2.3.1. Theoretical and Analytical Framework for This Study

Aligned with the framing of the overarching study, the CFIR was used as an analytical lens to support exploration of “what works where and why” [23]. In this study, the CFIR was applied to identify (a) outer setting factors beyond the immediate organizational context, and (b) inner setting factors within healthcare organizations, that shape the career trajectories of individual women nurses and midwives.

System‐level opportunities were defined as influences arising from the broader external environment (CFIR outer setting), including health policy, legislation, industrial agreements, professional regulation, funding structures, and societal perceptions of nursing, midwifery, leadership, and gender. Organizational‐level opportunities were defined as influences within healthcare organizations (CFIR inner setting), including organizational culture, workforce policies, leadership practices, recruitment and promotion processes, professional development opportunities, and workplace flexibility.

The CFIR is useful for identifying the multilevel factors that shape implementation, but it provides limited insight into how access to resources, support, information, and opportunities influences individual agency and career progression. To address this, we drew on Kanter’s structural empowerment theory [32, 33]. Structural empowerment refers to employees’ access to opportunities, information, support, and resources, as well as formal and informal sources of power [32]. In this study, it helped explain how organizational and system factors influence nurses’ and midwives’ progression into leadership roles.

Kanter’s framework provides insight into how contextual factors translate into individual experiences of empowerment or disempowerment. Together, the CFIR and Kanter’s structural empowerment theory enabled exploration of *what works, where, and why* (CFIR), and *how structural conditions enable or constrain individual agency* [32]. This dual approach is especially valuable when examining gendered career trajectories in nursing and midwifery, where both systemic and personal factors are intertwined.

Data from nurses and midwives were extracted from the primary dataset. Interview data and survey data were analyzed separately, with qualitative data analyzed thematically and quantitative data analyzed descriptively using categorical data in Qualtrics.

Template analysis was used to code qualitative interview and survey data. A provisional coding framework was developed both inductively and deductively, guided by the study’s aim, the CFIR, and structural empowerment theory, while remaining open to new insights [34]. Template analysis was chosen for its clear, systematic, and flexible approach to data analysis. The coding template remained adaptable throughout analysis, incorporating new themes, eliminating existing ones, and adjusting the scope and titles of the themes. Codes were organized hierarchically, with higher‐level codes representing overarching themes and lower‐level codes highlighting specific constituent themes. Initial coding was conducted by co‐author MP using Dedoose software [24], followed by discussions with co‐authors BG, IA, and KJ to finalize themes.

Quantitative survey data were analyzed using descriptive statistical analysis, specifically frequencies and percentages. Data analysis was performed using the Qualtrics software reporting tool [25].

Integration of qualitative and quantitative findings occurred during interpretation and reporting using a weaving narrative approach [35]. Qualitative themes and quantitative survey findings were compared side‐by‐side to identify convergence, divergence, and complementarity. Convergence occurred when findings aligned across datasets, divergence when different issues or priorities were emphasized, and complementarity when one dataset provided additional context or explanation for the other. Related findings were grouped into overarching themes to produce an integrated interpretation of opportunities for advancing women nurses and midwives into leadership. Findings were reported in accordance with the Guidelines for Conducting and Reporting Mixed Research in the Field of Counselling and Beyond [36].

### 2.4. Research Team and Reflexivity

Interviews were conducted by three experienced qualitative researchers with degrees in nursing (KJ), science (IA), and psychology (MM). No prior relationship existed between the participants and researchers. Co‐authors MP, BG, IA, and KJ actively participated in data analysis, holding frequent meetings to discuss data interpretation. MP and KJ shared their expertise in nursing and qualitative research, BG brought extensive experience in qualitative analysis, and IA provided specialized knowledge in intersectionality and qualitative research.

### 2.5. Ethical Approval and Consent

Ethical approval for this research was granted by the Monash University Human Research Ethics Committee (Project ID 25097). Informed consent was obtained from all participants before they engaged in any part of the research.

## 3. Results

### 3.1. Participant Characteristics

The participant numbers in the overarching study are noted in Figure 1. This substudy reports findings from interviews with healthcare leaders who were nurses and/or midwives (*n* = 25), with a median interview duration of 49:47 (range 27:25–67:27) minutes, and survey responses from health service employees who were nurses and/or midwives (*n* = 158) (Tables 1 and 2). Interviews were conducted between September 2021 and February 2024. Surveys were conducted between February 2022 and August 2024.

**TABLE 1:** Interview participants’ demographic characteristics.

Characteristic	Frequency (*n*)	Percentage (%)
Site (*n* = 25)		
Site 1	10	40
Site 2	2	8
Site 5	6	24
Site 6	7	28
State of residence (*n* = 25)		
Queensland	7	28
Victoria	18	72
Gender (*n* = 25)		
Women	23	92
Men	2	8
Occupation (*n* = 25)		
Nurse	23	92
Midwife	2	8
Leadership level (*n* = 25)		
Senior level (directors, executive general managers, and senior clinicians/clinical nurse consultants)	5	20
Middle level (departmental managers and unit heads)	8	32
Entry level (assistant nurse managers and nurse managers)	11	44
Highest level of formal education (*n* = 9)^†^		
Graduate diploma or graduate certificate level	4	44
Master’s degree level	5	56
Cultural background (*n* = 11)^†^		
Australian	7	64
New Zealander	1	9
Anglo‐European	2	18
Asian	1	9
Age, years (*n* = 10)^†^		
40–44	1	11
50–54	4	44
55–59	3	33
60–64	2	22

^†^Education level, cultural background, and age were collected as optional demographic items; denominators reflect only participants who provided this information (*n* = 9, *n* = 11, and *n* = 10, respectively), not the full sample of *n* = 25.

**TABLE 2:** Online survey participants’ demographic characteristics.

Characteristic	Frequency (*n*)	Percentage (%)
State of residence (*n* = 156)		
Queensland	127	80
Victoria	31	20
Occupation (*n* = 158)^†^ ^,^ ^‡^		
Nurse	146	92
Midwife	15	9
Nurse midwife	3	2
Hospital management	5	3
Researcher (nonuniversity)	1	1
Nondirect patient care	5	3
Other (ambulatory outpatient department, community mental health, and manager)	3	2
Gender (*n* = 156)		
Woman	145	93
Man	6	4
Prefer not to say	5	3
Age, years (*n* = 156)		
Younger than 25	7	4
25–29	11	7
30–34	12	8
35–39	29	19
40–44	18	12
45–49	21	13
50–54	30	19
55–59	14	9
60–64	13	8
65 or older	1	1
Indigenous Status: Australian Aboriginal and/or Torres Strait Islander person (*n* = 156)		
No	146	94
Yes	7	4
Prefer not to say	3	2
Years at current organization (*n* = 158)^†^		
Less than 5 years	46	29
5–10 years	31	20
10–15 years	42	27
15–20 years	18	11
20 years or more	21	13
Cultural background (*n* = 156)		
Indigenous Australian	4	2
Australian (excl. Indigenous Australian)	101	60
New Zealander (not Māori)	7	4
Māori, Melanesian, Papuan, Micronesian, and Polynesian	3	2
Anglo‐European	28	17
South‐East European	2	1
South‐East Asian	10	6
Southern and Central Asian	2	1
North American	2	1
Sub‐Saharan African	2	1
Unsure	1	1
Prefer not to say	4	2
Other cultural/ethnic group (please specify: Scottish, South African)	2	1
Highest level of formal education (*n* = 156)		
Certificate III or IV	1	1
Advanced diploma or diploma	5	3
Bachelor degree (including honors)	46	29
Graduate diploma or graduate certificate level	40	26
Master’s degree level	61	39
Doctoral degree level	3	2
Current employment arrangement (*n* = 156)		
Full‐time	84	54
Part‐time	67	43
Casual	3	2
Contract work	2	1
Career stage (*n* = 156)		
Recent graduate, less than 1 year of experience	3	2
Professional/healthcare provider, up to 5 years’ experience	16	10
Professional/healthcare provider, 5+ years’ experience	41	24
Long‐time technical expert/professional/healthcare provider without formal leadership/management role, 10+ years	37	22
First‐time leader/manager—in this role less than 6 months	10	6
Leader/manager, up to 5 years	22	13
Leader/manager, 5+ years	30	18
Senior executive/leader/manager	6	4
Other (please specify: education, leadership team under manager, clinical nurse have worked as Clinical Nurse Consultant and Nurse Unit Manager)	3	2

^†^Denominators vary across items (*n* = 156 or *n* = 158) and reflect the number of valid responses received for that survey item, not the full respondent sample.

^‡^Respondents could select more than one occupation category; percentages therefore sum to more than 100%.

Findings are presented using an integrated mixed‐methods approach, with points of convergence, divergence, and complementarity between qualitative and quantitative data highlighted throughout the themes.

### 3.2. System‐Level Opportunities

Seven themes and twenty subthemes were identified, highlighting system‐level and organizational‐level factors that offer strategic opportunities to advance women nurses and midwives into healthcare leadership roles. These were mapped to CFIR domains [22] and structural empowerment theory [32] (Table 3) and are illustrated in Figure 2.

**TABLE 3:** Summary of illustrative quotes.

CFIR construct	Theme	Structural empowerment framework	Subtheme: opportunity for strategic improvement	Illustrative quotes
Outer setting (system‐level)	1. Reform legal agreements	Access to opportunities	Revise Enterprise Bargaining Agreements to enable: 1.1. Pay equality for nursing and midwifery leaders 1.2. Flexibility, including in roles across clinical, research, and leadership, for women leaders in nursing and midwifery	*“[Enterprise Bargaining Agreements]. We just had to look at what does [the Enterprise Bargaining Agreement] say about managers, it’s obviously full-time. Then, of course, that’s it, full stop. You can’t compete with that. You can’t compete with that… other companies [small health services] that are changing this… when big organizations, like hospitals, are going to catch up with this [Enterprise Bargaining Agreement].”* **(Participant 1_9)** *“If you want to go into management or education or research, these are the only pathways for nurses…”* **(Participant 1_9)** *“there is no formal pathway and I think that’s something we really do miss.”* **(Participant 6_11)**
	2. Challenge perceived hierarchies within professional roles	Informal power and access to opportunities	2.1. Strengthen governance structures to include nursing and midwifery voices at strategic levels	*“The other dynamic that is difficult for nursing is that doctors have a lot of influence that nurses don’t… that’s very evident and can again be difficult to navigate in a role like mine where it’s a senior role and I’m trying to influence decisions being made primarily by a medical workforce… that’s a difficult dynamic anyway with that power dynamic in the medical profession and hospital administration.”* **(Participant 2_21)**

Inner setting (organization‐level)	3. Build leadership capacity through career pathways and inclusive organizational policies and processes	Access to opportunities, access to support, access to resources, and access to information	3.1. Workplace flexibility	*“We do short shifts, we do alternate start times…”* **(Participant 1_19)** *“I have nurses that would want to take on extra roles, but being mothers, and their partners working, themselves working and childcare… if you’re in a clinical role, that is hard and limiting for them.”* **(Participant 1_8)**
			3.2. Enable part‐time leadership roles and job‐sharing arrangements	*“You could look at it being a shared role between two people… or it could be like a 0.8 position…”* **(Participant 1_15)** *“but if I think at a nurse unit manager level, [SITE 1] has been very clear that it’s a Monday to Friday, it’s a 5-day a week…”* **(Participant 1_19)**
			3.3. Support workforce re‐engagement and leadership development postparental leave	*“getting a breastfeeding room and a designated fridge. One of my [Associate Nurse Unit Managers] pumps and puts her milk, and it because it’s a bodily fluid it has to be in a special [designated fridge], so they’ve created a breastfeeding room and a designated fridge. That’s taking into a fact that women come back after having babies and need somewhere to do that [breast milk expressing]…”* **(Participant 5_1)** *“keeping in touch, and the returning to work scenario so that people that have been out of the workforce, coming back in… I think there’s 10 working days where they can tap into keeping in touch and learning the role again.”* **(Participant 1_19)**
			3.4. Policies that promote equity in recruitment and promotion	*“They don’t advertise those roles. They tap people on their shoulders. It’s usually a male, it’s usually a doctor. And it’s usually a white male…”* **(Participant 6_7)** *“people are more inclined to actually recruit someone similar to them in their thinking and their approach. I see it with one of my direct reports that seem to recruit people similar to her, and she’s not a performer, and the staff don’t perform. So, I always ensure that I have someone on that panel that can actually counteract that. I think you have to have a really good panel selection to counteract that bias. And that’s why we always have an independent person on it in that respect as well.”* **(Participant 6_7)**
			3.5. Address gendered patterns of promotion	*“…young men coming in doing nursing, and they don’t stay in the profession of nursing, they kind of rise through the ranks…”* **(Participant 1_13)** *“…she has to be perfect of perfect to get through as against a guy can be good enough and okay and get through…”* **(Participant 5_4)** *“I think it’s an unspoken bias… bias against women… I think women have to prove themselves… people have to see the results to get credibility…women have to work harder to get there.”* **(Participant 1_14)**
			3.6. Transition pathways from clinician to manager/leader	*“We’re running a transition to [Associate Nurse Unit Manager]. What does an [Associate Nurse Unit Manager] do… There needs to be more opportunity for people to look at these types of things, whether formally or informally, and have the opportunity to act up into a different position… There needs to be transparency for people to have these opportunities.”* **(Participant 5_5)**
	4. Foster inclusive organizational cultures through awareness and engagement	Access to opportunity	4.1. Elevate visibility of role models and representation	*“celebrating those types of achievements from women, it’s powerful and it’s certainly – you go to those forums … it’s pretty inspiring”* **(Participant 1_19)** *“I think it allows [women] to feel an equal. It allows them to feel worthy, worthy of their positions. It allows them to feel successful. It increases their confidence and their passion to strive for bigger and better, and it opens up more opportunities. It increases their motivation to take on even bigger challenges that they may have not even thought of. And not feel scared that it’s out of their league because it’s not a woman’s position to be in, basically.”* **(Participant 1_14)**
			4.2. Challenge cultural forms to enable inclusive leadership	*“I have noticed that the women are over-spoken by men…The more and more you see, it still happens. I’ve been quite assertive in a meeting, but it’s been seen as aggressive, because a man can be aggressive, and it’s not seen as aggressive. It’s really interesting.”* **(Participant 1_18)** *“I’m used to doing a cultural survey every three, 4 years, but [Site 5] do one annually. It’s not just a tick box exercise, they do the culture survey, the staff that want to participate, and then they action them.”* **(Participant 5_1)**
			4.3. Strengthen equity through diversity and inclusion education	*“I think it’s really helped create an awareness that we are all equal and everybody has the right to be who they say they are… the diversity, inclusion, the respect and kindness training has certainly started to – I think it’s starting to create that cultural shift…”* **(Participant 1_14)**
			4.4. Increase policy awareness and accessibility	*“our equal opportunity policy…and the fundamental 5Cs that we have that underpin that. But also, the [online learning]. It made me think what’s important to them, rather than what’s important to me? It really helped me get to provide them with what they needed rather than focus on what I needed to achieve.”* **(Participant 1_14)**
	5. Expand mentoring and networking opportunities	Access to opportunities, access to support, access to resources, and access to information	5.1. Facilitate formal and informal approaches for women in nursing and midwifery to identify mentors and participate in and build effective networks 5.2. Invest in coaching and mentoring to support leadership readiness	*“I would be able to ask [my internal mentor], for example, how did you – you’re part of [SITE 1], have you ever experienced, how should I? Ask her more from an organizational-lived experience. My external mentor was able to give me very kind of strategic, broad reflections.”* **(Participant 1_14)** *“For health network, it’s often about validating processes, systems, kind of understanding your industry… for example, there’s a group of women that we catch up… and it’s very much about that kind of support, peer – I guess it’s almost puffing up your feathers a little bit, that sometimes you don’t get in your own organization, and giving you some tips and tricks at how to manage some of those intricacies…”* **(Participant 1_13F)**
	6. Enhance leadership development through accessible, tailored training	Access to opportunities, access to support, access to resources, and access to information	Develop Women Nursing and Midwifery Leaders by Enhancing Professional and Leadership Development Opportunities: 6.1. Embed leadership development into workforce planning, with clear pathways from clinical to leadership roles 6.2. Ensure training is flexible and accessible, especially for part‐time staff 6.3. Promote awareness of programs	*“They’re running sessions for nurse managers, mid-level, and giving us further opportunities to develop other skills in the area. That’s really helpful for us if we were looking at advancing up into the next level of directorship…”* **(Participant 5_5)** *“I think, clear pathways. [SITE 1] are really good like that with all their leadership program and pathways. I think they break the stereotype… so that pathway’s really important.”* **(Participant 1_18)** *“There are a couple of staff who want to work permanent nights, and have been told that they have to do dayshifts to keep their skills up…”* **(Participant 1_11)** *“…being able to make sure that one size isn’t going to fit all…”* **(Participant 1_13)**
	7. Embed equity and transparency in recruitment and promotion	Access to information and access to opportunity	7.1. Promote fair and transparent recruitment by standardizing processes, diversifying panels, using blind recruitment, and openly advertising all roles 7.2. Bias‐reduction training, monitoring gender representation, and eliminate informal selection practices	*“…with management roles, we should actually be taking names away and just do like a blank almost… I don’t know how exactly we’d do it, but take away names, genders, voice…”* **(Participant 5_4)** *“When positions become available, especially temporary positions, there’s always that [Expression of Interest] process that would be followed… there’s always someone who sits on those panels from outside the area. And there’s always at least three people on the panels, usually multidisciplinary as well…”* **(Participant 6_8)**

Abbreviation: CFIR, Consolidated Framework for Implementation Research.

**FIGURE 2:**
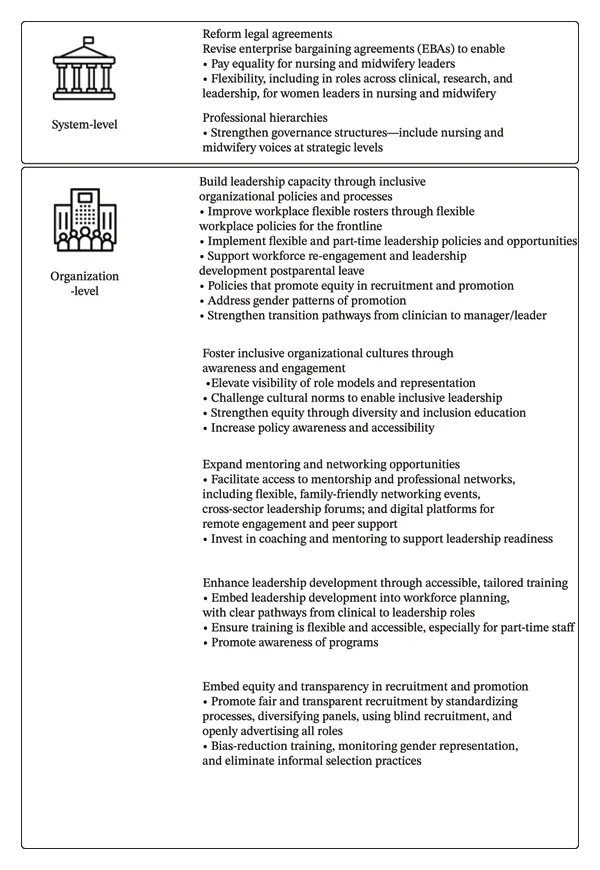
Opportunities for strategic improvement for advancing women nurses and midwives into healthcare leadership.

#### 3.2.1. Reform Legal Agreements

Participants identified EBAs as a key lever for change. While EBAs were reported to include provisions for flexible work arrangements, their practical application to leadership roles was limited. Interviewees suggested that incorporating part‐time and flexible arrangements into leadership roles within EBAs could reduce barriers to advancement, while balancing other responsibilities. While some smaller health services were beginning to challenge the prevailing assumption that leadership roles must be full‐time, larger health services were perceived to have greater structural and cultural barriers to implementing flexibility at senior levels for nurses. Consequently, EBAs were widely viewed as providing structured guidance for healthcare managers, particularly in navigating flexible work arrangements within clinical settings; however, their application to leadership roles remained limited and inconsistent.

Beyond workplace flexibility, participants identified limited career pathways and opportunities to expand nursing scope of practice as important areas for reform. Flexibility of roles across clinical, research, and leadership under the one EBA was also an identified opportunity, yet this was not as flexible in the nursing and midwifery workforce.

Building pay equity into legal agreements was also identified as an important opportunity to attract and retain women nurses and midwives into leadership roles. Nursing leaders highlighted a disparity between the responsibilities, workload, and remuneration associated with nursing leadership compared to other disciplines and sectors. Pay inequity was perceived to devalue the contribution of nurses in leadership positions.

“If I was in another industry, with the responsibility I have and the commitment and the workload, I would be on a very different pay scale” [Participant: 1_13]

#### 3.2.2. Challenge Perceived Hierarchies Within Professional Roles

Participants across career stages reported that opportunities for advancement into healthcare leadership were constrained by entrenched professional hierarchies. Nurses and midwives were reportedly positioned as subordinates to medical officers, who were perceived to hold both formal and informal leadership power. As one participant described:

“the doctors are seen as the leaders, and then we’re under them” [Participant: 1_11]

This perception was reinforced by survey findings, with 44% (*n* = 63) of respondents believing leadership roles were more accessible to medical professionals and 46% (*n* = 66) agreeing that career advancement opportunities were limited by their profession. Together, these findings highlight the influence of discipline‐related bias on leadership progression and the need for greater recognition of the leadership capacity of nurses and midwives.

Interview participants further described these professional hierarchies as particularly evident in the private sector, where medical officers were perceived to hold greater influence because of their role in attracting patients and generating revenue. Many senior nurse leaders reported having limited influence in organizational decision‐making, which was often dominated by the medical workforce. This imbalance in informal power and influence was viewed as a significant barrier to leadership advancement.

Convergence was evident across the qualitative and quantitative data, with both datasets indicating that professional hierarchies continue to shape access to leadership opportunities and decision‐making influence for nurses and midwives. Strengthening governance structures to increase nursing and midwifery representation at strategic levels was identified as an important opportunity to address these imbalances.

### 3.3. Organization‐Level Opportunities

#### 3.3.1. Build Leadership Capacity Through Inclusive Organizational Policies and Processes

A number of policies, processes, and practices were identified as important. These included workplace flexibility, part‐time leadership roles and job‐sharing arrangements, support when transitioning back to work after parental leave, equitable recruitment and promotion policies, support during the transition from clinician to manager, secondment opportunities, and clear leadership pathways.

Both leaders and employees identified these organizational supports as important enablers of leadership progression, demonstrating convergence across the qualitative and quantitative findings.

##### 3.3.1.1. Workplace Flexibility

Workplace flexibility was consistently identified as a key organizational strategy for enabling women nurses and midwives to pursue and progress into leadership roles. Senior leaders described a range of flexible approaches that could be implemented at organizational levels, including advance rostering, permanent or self‐rostering options, and shorter shifts to accommodate caregiving responsibilities. Rigid scheduling practices, such as set 12‐h shifts and early morning start times (e.g., 7:00 am), were highlighted as significant barriers, particularly when they conflicted with parenting availability. In response, some partners had introduced alternative shift structures to better support staff with caring commitments. For those in leadership or management roles, flexibility extended to options, such as working from home, flexible hours, and adjusted start times, enabling greater balance between professional responsibilities and caring commitments. One respondent noted, “shift start and finish times should be more accommodating to school/childcare times,” reflecting a broader sentiment that flexible work arrangements are essential for retention and hence for enabling women to advance into and sustain leadership roles within the healthcare sector.

##### 3.3.1.2. Enabling Leadership Role Flexibility and Job‐Sharing Arrangements

Enabling leadership role flexibility and job‐sharing arrangements represents another key organizational strategy for enabling career progression for nurses and midwives, particularly for women returning from parental leave or relocating. Interview participants identified these flexible models as essential for retaining experienced professionals and supporting their transition and sustainability in leadership roles. However, survey data highlighted a significant gap in availability: 47% (*n* = 50) of respondents reported that job‐sharing options were not available in their workplace. These findings were complementary. While survey data identified the limited availability of flexible leadership arrangements, interview participants provided insight into the organizational norms and structural barriers that limited implementation of part‐time and job‐sharing leadership roles.

Senior leaders noted that many leadership positions, such as Nurse Unit Manager roles, were structured around full‐time availability, with some describing this requirement as a “hard‐and‐fast rule” and “not negotiable.” This rigidity was perceived as a systemic barrier to inclusive leadership pathways, often forcing aspiring nurse leaders to return to clinical roles, thereby interrupting their leadership trajectory and limiting advancement opportunities.

In contrast, participants noted that in medical roles, part‐time or fractional appointments were more commonly accepted for clinical, research, and leadership roles. This disparity underscores the need for healthcare organizations to re‐evaluate leadership role design and embed flexible models into workforce planning and implementation of updated EBAs. Doing so would not only support gender equity in leadership but also strengthen organizational capacity by retaining skilled nurse and midwife leaders.

Supporting clinical nurses in transitioning to leadership roles requires clear guidance on role expectations, implementing structured preparation both formal and informal and establishing transparent processes for access to acting or developmental opportunities. Senior leaders also highlighted the value of secondment opportunities that allow nurses and midwives to act in higher leadership roles on a temporary basis. When combined with clearly defined leadership career pathways, these opportunities were considered effective in building leadership capacity and supporting progression into senior roles.

##### 3.3.1.3. Supporting Workforce Re‐Engagement and Leadership Development Postparental Leave

Supporting workforce re‐engagement and leadership development postparental leave was also identified as a key strategy for enhancing women’s capacity to remain engaged in the workforce, progress toward, and remain in leadership roles. Senior leaders reported the provision of dedicated breastfeeding rooms equipped with privacy features, fridges, and power outlets as important workplace accommodations. Additional supportive measures included extended break times for breastfeeding mothers, on‐site childcare services, and partnerships with local childcare providers to align with shift schedules. These supports were viewed as critical enablers for balancing caregiving responsibilities with professional advancement. Initiatives, such as “keep‐in‐touch” days and ongoing access to professional development during parental leave, were also highlighted as effective strategies for maintaining engagement and continuity in career progression.

##### 3.3.1.4. Policies That Promote Equity in Recruitment and Promotion

Policies that promote equity in recruitment and promotion, including equal opportunity frameworks and strategies aimed at “recruiting to diversity” [Participant: 1_8], were identified as essential for increasing the representation of women, nurses, and individuals from culturally diverse backgrounds in leadership. This perspective was supported by nurse and midwife employees, with 70% (*n* = 86) agreeing that the best candidate for the role should be selected based on their relative opportunities and potential for success, rather than solely on traditional metrics of experience or tenure.

Support for affirmative action was also evident, with 66% (*n* = 81) endorsing its role in advancing gender equity in healthcare leadership. Notably, only 9% (*n* = 11) viewed affirmative action as unfair, while nearly half (49%; *n* = 61) disagreed with the notion that such measures are unjust. These findings suggest broad support for targeted strategies to correct historical imbalances and promote inclusive leadership pathways. Qualitative and quantitative findings aligned in emphasizing the importance of equitable recruitment and promotion practices. While leaders highlighted organizational strategies to increase representation and fairness, employees expressed strong support for approaches that recognize potential and address historical inequities.

##### 3.3.1.5. Address Gendered Patterns of Promotion

Despite nursing and midwifery being women‐dominated professions, participants reported persistent gendered patterns of promotion that disproportionately favor men in this field. Participants described a persistent “glass ceiling” [Participant: 2_21] that limits women’s advancement, with men nurses often progressing more rapidly into mid‐ and senior‐level roles—even when they possess less experience or fewer qualifications. Survey data reinforced this concern, with 45% (*n* = 62) of respondents agreeing that men in nursing are more likely to be promoted into leadership roles than women. Participants attributed this trend to societal perceptions and structural advantages that position men as natural leaders, while women are often required to demonstrate sustained performance and tangible outcomes to establish credibility.

Leaders observed that men frequently transition from bedside roles into leadership positions, bypassing traditional pathways. In contrast, women reported needing to adapt their behavior to compete with male colleagues, rather than being recognized for their distinct leadership strengths. These dynamics were perceived to perpetuate exclusionary practices and reinforce societal gender biases within healthcare organizations.

##### 3.3.1.6. Strengthen Transition Pathways From Clinician to Manager/Leader

The transition from clinical roles to leadership positions was widely perceived by participants as requiring strong organizational support and targeted leadership development. Senior leaders emphasized that while clinical competence is essential, it does not automatically translate into effective leadership. The prevailing “culture of working your way up” [Participant: 6_8] where leadership roles are assumed through tenure rather than talent, aptitude, and structured development, were considered inadequate. Some participants reported access to structured transition supports, such as acting roles, secondments, or formal leadership development opportunities or pathways. Where available, these opportunities were seen as highly beneficial in building confidence and competence. Senior leaders also highlighted the importance of investing in digital tools and resources to enhance leadership capability. Platforms, such as virtual meeting software, online dashboards, and access to professional memberships, were identified as valuable in cultivating core leadership competencies. By integrating these technologies into daily workflows, leaders can build their adaptability, improve communication effectiveness, and foster continuous professional growth. Interview data helped explain patterns observed in survey responses, providing detailed insight into the organizational supports, transition processes, and development opportunities required to facilitate progression from clinical practice into leadership roles.

#### 3.3.2. Foster Inclusive Organizational Cultures Through Awareness and Engagement

Promoting awareness of gender equity challenges and organizational policies was consistently identified as a key strategy for cultivating a culture of inclusion, engagement, and opportunity. Senior leaders emphasized the importance of transparency and visibility in equity efforts, noting that publicizing both achievements and ongoing challenges can motivate broader workforce participation. Some leaders advocated for gender bias and cultural awareness training to address unconscious bias, particularly as it affects women from ethnically diverse backgrounds, “it’s understanding there is what you do know, but there’s also that unconscious bias or that unconscious behavior that people aren’t aware of” [Participant: 1_8]. Survey data supported the emphasis on inclusion, with 71% (*n* = 102) of respondents agreeing that women were well represented in leadership at the team or unit level, yet only 43% (*n* = 64) perceived balanced gender representation at the organizational level, highlighting a gap in visibility and influence at senior levels. Leader and employee perspectives were closely aligned, highlighting the importance of visibility, representation, and inclusive workplace cultures, while also identifying continuing gaps in representation at senior leadership levels.

##### 3.3.2.1. Elevate Visibility of Representation and Role‐Modeling

Senior leaders acknowledged progress in women’s representation at early and mid‐level leadership roles but noted persistent underrepresentation at executive and board levels. Participants stressed the importance of visible role models and storytelling, such as sharing journeys from senior clinical roles to executive leadership, as a way to inspire and normalize women’s leadership. Organizational initiatives, such as annual staff awards, which recognize long service and excellence in nursing, were viewed as important for acknowledging the contributions of nurses and midwives. Events, such as the Women’s Leadership Summit, Women’s Forum, and Women in Healthcare Lunch, were seen as empowering. These initiatives were valued for recognizing contributions and celebrating women’s achievements, reinforcing a sense of belonging and aspiration.

##### 3.3.2.2. Challenge Cultural Norms to Enable Inclusive Leadership

Despite progress, several senior leaders described ongoing cultural challenges, including women being “over spoken by men,” the persistence of a “boys’ club” mentality, aggressive behaviors in meetings, and exclusion from informal networks. To foster inclusivity, it is essential to address and overcome persistent traditional cultural and societal norms. These dynamics were seen to limit access to leadership opportunities and reinforce stereotypes, underscoring the urgent need for organizational cultural transformation to create truly inclusive and supportive leadership environments. Leaders called for intentional efforts to dismantle these barriers, including regular evaluation of organizational culture through tools, such as annual staff surveys.

##### 3.3.2.3. Strengthen Equity Through Diversity and Inclusion Education

Mandatory training, policies, and strategies on cultural diversity, nondiscrimination, and inclusion were viewed as essential for embedding equity into workplace culture. While some organizations had implemented annual training, leaders emphasized the need for consistent messaging and reinforcement. Proposed strategies included tailored approaches to accommodate cultural and religious practices, inclusivity campaigns, inclusive recruitment and promotion policies and practices, and visible recognition of workforce diversity. As one leader stated, organizations must “continue to talk the language, be consistent in their message, and promote it more” [participant: 1_14].

##### 3.3.2.4. Increase Organizational Policy Awareness and Accessibility

Flexible work, parental leave, equal opportunity, and inclusive recruitment policies were considered foundational to advancing women nurses and midwives into leadership. While most employees reported awareness of these policies, senior leaders stressed the importance of ensuring they are accessible, well‐communicated, and actively implemented. Survey responses indicated strong support for family‐friendly policies, with many respondents citing them as critical enablers of career progression.

#### 3.3.3. Expand Mentoring and Networking Opportunities

Mentoring was consistently identified by senior leaders as a critical enabler of leadership progression, with participants describing both internal and external mentoring relationships that supported their development. These relationships varied in formality and gender composition, ranging from formal structured programs to informal or organically developed connections. Mentoring was perceived to provide essential guidance, confidence, and exposure to leadership thinking, particularly for women navigating complex career transitions.

Despite its recognized value, survey data revealed limited engagement with mentoring opportunities. A majority of respondents (59%, *n* = 64) had not previously participated in mentoring. Among those who had, most were mentored internally (64%, *n* = 29), while only 11% (*n* = 5) had access to external mentors, and 20% (*n* = 9) experienced both. Notably, 89% (*n* = 40) of respondents sought mentoring from women, suggesting a strong preference for gender‐congruent support in leadership development.

Awareness of formal mentoring programs was also low: 71% (*n* = 63) of employed nurses and midwives were unaware of any formal mentoring program within their organization, and only 16% (*n* = 14) had accessed such programs.

Senior leaders also emphasized the importance of professional networking, both within and beyond the healthcare sector, as a key strategy for leadership development. From the employee perspective, 54% (*n* = 60) reported access to networking opportunities within their organization or affiliated networks, while 43% (*n* = 48) identified opportunities outside their organization. Findings from both datasets reinforced the importance of mentoring and networking for leadership development. While leaders emphasized their value, employees reported limited access and low awareness of available opportunities, highlighting a significant unmet need.

#### 3.3.4. Enhance Leadership Development Through Accessible Training

Leadership development was widely recognized by senior leaders as a critical enabler for advancing women nurses and midwives into leadership roles. Participants emphasized the importance of offering flexible and accessible leadership training opportunities, tailored to accommodate diverse work schedules, caregiving responsibilities, and career stages. However, survey data revealed variability in access: While 46% (*n* = 56) of respondents agreed that their organization provided flexible training options, 35% (*n* = 43) disagreed, highlighting inconsistent availability across settings.

Several senior leaders noted a lack of structured leadership development, with many nurses and midwives expected to “learn on the job” without formal preparation. This was particularly evident in early‐career leadership roles, where ad hoc learning often replaced intentional development. Both general leadership programs and those specifically designed for women were viewed as valuable, though gender‐specific initiatives appeared to be limited. Where available, these programs were seen to foster confidence, communication, and strategic thinking among emerging leaders.

Organizational initiatives, such as internal and external training programs, scholarships, funding support, and ongoing professional development, were frequently cited as essential for building leadership capacity. Yet, awareness of these opportunities varied: Only 26% (*n* = 28) of employees reported being well aware of available programs, while 50% (*n* = 55) were somewhat aware, and 24% (*n* = 26) were not aware at all. Among those who had participated, 79% (*n* = 44) found the programs valuable for enhancing their skills and professional growth.

While 67% (*n* = 73) of employees agreed that their organization supported their participation in professional development, open‐ended responses revealed barriers to accessibility, including requirements to attend training outside paid hours and the exclusion of part‐time staff from development opportunities.

Senior leaders highlighted the importance of empowering women through coaching, performance planning, and career counseling, with a focus on “building their competence, building their belief in themselves” [Participant: 5_8]. They also reiterated the organization’s responsibility to actively support women’s leadership development by “giving them the skills, knowledge, and supports” [Participant: 1_13] necessary to succeed. Survey responses revealed variability in access to leadership development opportunities, and interview findings helped explain these experiences by highlighting limited structured pathways and continued reliance on informal learning approaches.

#### 3.3.5. Embed Equity and Transparency in Recruitment and Promotion

Senior leaders identified a range of organizational strategies aimed at improving equity in recruitment, selection, and promotion processes. These included the following:

• Enhancing diversity on recruitment panels by including interviewers from outside the immediate team or department to reduce bias and broaden perspectives.
• Implementing blind recruitment practices, such as removing personal identifiers (e.g., names and gender), to promote fairness and reduce unconscious bias.
• Enhancing transparency by clearly advertising roles and expressions of interest, particularly for temporary and acting positions, to ensure equitable access.

Despite these efforts, employee perceptions revealed ongoing concerns. Less than half agreed that their organization had transparent processes for recruitment (41%, *n* = 46), selection (36%, *n* = 39), or promotion (29%, *n* = 32). Additionally, 58% (*n* = 63) perceived leadership selection as biased, and 71% (*n* = 78) believed organizations should actively monitor and assess gender equity as part of continuous improvement efforts.

Participants described ongoing gendered patterns of promotion, with men perceived as more likely to promote other men. This dynamic was often referred to as the “boys’ club” or “white male club” [Participants: 1_8, 1_20, 6_7, 5_4], reflecting informal networks that influence leadership appointments. Some organizations were reported to rely on informal practices, such as “shoulder tapping,” where individuals were invited to apply or assume leadership roles based on personal relationships or networks, rather than on merit. One leader observed, “There are a lot of people who rub up the right trees… they sort of get these key roles just because of who they know” [Participants: 1_11]. In some cases, leadership roles were not publicly advertised, and individuals were appointed to acting positions for extended periods without open selection, limiting access for others and reinforcing inequities. Together, the qualitative and quantitative findings underscored the importance of transparent and equitable recruitment processes. Leaders highlighted strategies for reducing bias and strengthening fairness, while employees described ongoing concerns regarding selection practices and promotion decisions.

## 4. Discussion

This study explored the perspectives and experiences of nurses and midwives to identify strategic system‐level and organizational‐level opportunities for advancing women nurses and midwives into healthcare leadership. Drawing on the CFIR and Kanter’s structural empowerment theory, the findings highlight how access to information, support, resources, and opportunities shape leadership trajectories for women in nursing and midwifery.

### 4.1. System‐Level Strategic Opportunities

System‐level reforms were identified as critical enablers of leadership advancement for nurses and midwives. Revising EBAs to support pay equity, flexible work arrangements, and role diversity emerged as a key opportunity. Although EBAs provide a formal mechanism through which nurses and midwives access career opportunities, current agreements often lack provisions for part‐time leadership positions and hybrid roles that combine clinical practice, leadership, research, and education. This limits career progression, particularly for women balancing professional and caregiving responsibilities, and represents an ongoing barrier within the Australian nursing and midwifery workforce [37].

Although EBAs outline distinct pathways for clinicians, managers, researchers, and academics, they rarely support movement across these roles or the development of integrated career pathways. Their traditional emphasis on fixed classifications and rigid award structures may constrain the flexibility increasingly required within contemporary healthcare workforces [37, 38]. Comparisons with medical EBAs, which support clinician‐researcher and clinician‐educator models, suggest nursing and midwifery could benefit from similar approaches [38, 39]. Beyond supporting career progression, these integrated roles may also strengthen clinical leadership, innovation, and workforce sustainability.

However, reform is not without challenges. Introducing flexible leadership arrangements, hybrid roles, or revised classification structures may require negotiation between employers, employees, professional associations, and governments, particularly within resource‐constrained health systems. These competing priorities are likely to influence the pace and extent of reform.

Participants also identified funding support as an important facilitator of leadership advancement. Unlike the medical profession, which often benefits from dedicated continuing medical education entitlements [40], nurses and midwives frequently have limited access to funded leadership and professional development opportunities, requiring many to self‐fund their development unless scholarship support is available.

Nursing professional organizations play an important role in advocating for structural and policy reforms that support workforce equity and leadership advancement [41]. Given the profession’s historical, social, and political context, including its gendered nature and longstanding dominance of medicine within healthcare systems, nursing organizations are well positioned to amplify the collective voice of nurses and influence policy agendas, workforce reform, and leadership development initiatives [41].

While EBAs are specific to the Australian industrial relations context, the broader principles identified in this study have wider relevance. Workforce policies that support flexible leadership roles, transparent career pathways, equitable remuneration, and leadership development opportunities are likely to be applicable across many healthcare systems. Although some recommendations are context‐specific, the underlying mechanisms for advancing nursing and midwifery leadership are likely transferable to other settings. Implementation would, however, need to be tailored to reflect local workforce structures, regulatory environments, and funding models.

Participants also highlighted entrenched professional hierarchies that position nurses and midwives as subordinate to medical professionals, constraining access to leadership opportunities and informal power. These findings align with our previous systematic reviews and the broader literature examining leadership and professional hierarchies in health care [4, 42–44].

These hierarchies are closely linked to longstanding perceptions of nursing and midwifery as professions primarily associated with caregiving rather than strategic leadership. Such identity narratives may limit recognition of nurses’ and midwives’ contributions to organizational leadership, policy development, and health system reform. Strengthening professional identity as both a clinical and leadership discipline may therefore support greater recognition of nurses and midwives as key contributors to organizational decision‐making and system transformation.

Study findings further indicate that nurses and midwives continue to encounter barriers when progressing to senior leadership and executive‐level positions. These roles not only offer greater influence and strategic decision‐making authority, but also provide opportunities to shape organizational priorities and health system reform. Representation of nurses and midwives at executive and board levels is particularly important given their trusted, patient‐centered perspective, professional expertise, and advocacy role in healthcare governance and policy development [45]. Addressing professional hierarchies through interdisciplinary leadership models and more inclusive governance structures may help rebalance influence and strengthen nursing and midwifery representation within strategic decision‐making forums.

Together, these findings suggest that system‐level policies, workforce structures, and professional hierarchies influence leadership advancement by shaping access to opportunities, resources, and informal power, key components of structural empowerment.

### 4.2. Organization‐Level Strategic Opportunities

At the organizational level, inclusive policies and practices were identified as essential for building leadership capacity. Flexible rostering, part‐time leadership roles, and structured re‐entry supports, especially following parental leave, were viewed as critical for maintaining career continuity and supporting progression to leadership positions. Inclusive organizational cultures were also seen as important, particularly when supported by awareness and engagement initiatives that promote equitable access to opportunities, information, and resources.

However, access to these supports was inconsistent, particularly within larger health services. Participants highlighted opportunities to strengthen mentoring and networking through family‐friendly networking events, cross‐sector leadership forums, and digital platforms that support remote engagement and peer connection.

Leadership development and career pathways emerged as key areas for strengthening. Participants reported limited access to structured transition programs from clinical to leadership roles, with many expected to learn management skills informally “on the job.” Embedding leadership development earlier in nurses’ and midwives’ careers and providing accessible, flexible training may improve leadership readiness, progression, and retention. This is consistent with emerging international midwifery leadership literature, which highlights the importance of supportive organizational environments, leadership development opportunities, mentoring, networking, and visible role models in enabling leadership progression [46]. Similar to our findings, these authors identified organizational and structural enablers as critical for strengthening leadership pathways within the profession.

Participants also highlighted the value of digital tools in supporting leadership development. Virtual meeting platforms, online professional development programs, performance dashboards, and digital networking opportunities were viewed as facilitating leadership capability and access to organizational resources. As healthcare systems increasingly adopt digital technologies and artificial intelligence–enabled decision‐support tools, digital literacy is increasingly recognized as an important leadership capability. Ensuring equitable access to digital capability development may therefore represent an additional strategy for strengthening future nursing and midwifery leadership pathways.

These findings suggest that organizations should embed leadership development into workforce planning, establish clear progression pathways from clinical to leadership roles, ensure training is accessible to part‐time and early‐career staff, increase awareness of available programs, and invest in coaching and mentoring to support leadership readiness.

Career pathways remain underdeveloped within nursing and midwifery. Current practice is hindered by the absence of standardized leadership role descriptions, clearly defined skill sets, and consistent frameworks to guide progression into leadership positions [47]. Entry‐level leadership positions, such as Clinical Nurse Specialist and Associate Nurse Unit Manager, are important stepping stones toward more senior leadership roles. Yet inconsistent titles, role descriptions, and locally determined recruitment processes may contribute to bias, favoritism, and limited role recognition.

Australian literature highlights that many advanced nursing titles are not protected, with more than 70 different designations identified across entry‐level nursing and midwifery leadership positions. These roles are often developed in response to local needs rather than through strategic workforce planning [47, 48]. This presents an opportunity for nursing organizations, colleges, and professional bodies to advocate for greater standardization and clearer leadership pathways [41, 47].

Workforce planning should move beyond staffing numbers alone and consider the skills, experience, and leadership capability required within the nursing and midwifery workforce [41, 49]. Such approaches may strengthen leadership development and retention while contributing to improved patient safety and quality of care [48].

Mentoring and networking were consistently identified as valuable but underutilized, with many participants reporting limited awareness of available opportunities. Expanding mentoring initiatives, particularly those designed to support women and culturally diverse staff, may help increase visibility, confidence, and leadership progression [16, 43, 50].

An intersectional perspective is important when considering leadership advancement in nursing and midwifery. Although many of the opportunities identified in this study are likely to benefit women broadly, leadership experiences are not uniform. Women nurses and midwives from ethnically diverse, migrant, Indigenous, or other minoritized backgrounds may encounter barriers that are not adequately addressed through gender‐focused initiatives alone. These may include discrimination, reduced access to influential networks, lower organizational visibility, and challenges linked to cultural expectations or migration experiences. Consequently, organizational strategies should be implemented with consideration of how intersecting identities influence access to opportunities and career progression.

Organizational culture also plays a central role in shaping leadership access [27, 43]. Participants described persistent gendered patterns of promotion, informal selection practices, and exclusion from influential decision‐making networks. Addressing these inequities requires organizations to formalize recruitment and promotion processes, monitor equity outcomes, eliminate informal selection practices, and strengthen bias‐reduction and inclusive recruitment strategies.

Together, these findings suggest that organizational factors influence leadership advancement by shaping nurses’ and midwives’ access to opportunities, information, support, and resources, reinforcing the central role of structural empowerment in leadership development.

### 4.3. Structural Empowerment and Leadership Advancement

This study, together with our previous systematic reviews and meta‐syntheses, highlights the substantial influence of system‐level and organizational‐level factors on leadership advancement for women nurses and midwives [4, 4, 16]. Opportunities, such as flexible leadership arrangements, leadership development programs, mentoring, networking, transparent recruitment processes, and clear career pathways, increase access to opportunities, information, support, and resources. Consistent with the Ability–Motivation–Opportunity (AMO) framework, these findings demonstrate how organizational conditions shape nurses’ and midwives’ capacity and opportunity to pursue leadership roles.

Kanter’s structural empowerment theory provides a useful lens to understand these findings. Kanter proposes that employees are more likely to feel empowered when they have access to information, support, resources, and opportunities [32]. Rather than relying solely on individual capability, leadership advancement is influenced by organizational environments that enable nurses and midwives to participate in decision‐making, exercise influence, and progress into leadership positions. Within this framework, power is associated with autonomy and competence rather than control, enabling individuals to contribute meaningfully to organizational goals [51].

However, access to these opportunities is unlikely to be experienced equally by all women. An intersectional perspective suggests that structural empowerment may be shaped by ethnicity, cultural background, migration history, and other social identities. Consequently, efforts to advance women nurses and midwives into leadership should consider intersectional inequities that may create additional barriers beyond gender alone.

These findings reinforce the need to move beyond individual‐focused approaches and address the organizational and system conditions that shape leadership opportunities for women nurses and midwives.

### 4.4. Strengths and Limitations

This mixed‐methods study has several strengths. It combines qualitative interviews and quantitative survey data from diverse healthcare settings across Australia, providing a rich understanding of opportunities to advance women nurses and midwives into leadership. The use of the CFIR and Kanter’s structural empowerment theory provides a strong theoretical foundation for examining structural influences on leadership advancement. The coproduction approach involving stakeholders throughout the research process strengthens the relevance and practical applicability of the findings. Importantly, the study identifies actionable system‐level and organizational‐level strategies that may inform policy and practice.

The primary dataset from which this substudy was drawn was designed to examine organizational strategies within broader healthcare leadership contexts rather than focus specifically on nursing and midwifery. As such, some nursing and midwifery‐specific opportunities may not have been fully captured.

Demographic representation within the qualitative sample was limited. Although 25 nursing and midwifery leaders participated in interviews, completion of demographic questions varied because participants were not required to answer all items. Consequently, demographic information was only available for some participants, limiting assessment of sample representativeness and subgroup experiences. Only 11 interview participants provided cultural background information, with limited representation from ethnically diverse groups. As a result, the findings predominantly reflect the experiences of participants from Australian and Anglo‐European backgrounds and likely do not fully capture the leadership experiences of ethnically diverse, migrant, Indigenous, or other minoritized nurses and midwives. The study was therefore unable to explore intersectional differences in depth.

The study did not collect detailed information on gender diversity, including transgender status or sex assigned at birth. Consequently, it was not possible to examine whether leadership experiences differed across diverse gender identities. References to men and women within the findings therefore reflect participant narratives and perceptions rather than independently verified gender identity categories.

Although this study focused on both nurses and midwives, the sample was predominantly comprised nurses. Despite recruitment efforts targeting both professions, participation from midwives was limited. Consequently, the findings may more strongly reflect nursing perspectives and may not fully capture experiences unique to midwives. Further research focused specifically on midwifery leadership pathways is warranted.

Finally, the study was conducted within the Australian healthcare context, including specific regulatory and industrial frameworks. This may limit the transferability of some findings, particularly those relating to EBAs and workforce regulation.

### 4.5. Implications for Policy, Practice, and Research

#### 4.5.1. Implications for Policy and Practice

System‐level reform should include policies and EBAs that support flexible, part‐time, and hybrid leadership roles integrating clinical practice, leadership, education, and research. Clear leadership pathways should be established through nationally consistent role titles, standardized position descriptions, and transparent progression frameworks for positions, such as Associate Nurse Unit Manager and Clinical Nurse Specialist. Recruitment and promotion processes should be transparent and supported by accredited leadership development, mentoring, and education programs. Continued policy advocacy by nursing organizations will be important in progressing these reforms.

At the organizational level, workforce planning should incorporate principles of structural empowerment by expanding flexible leadership models, formalizing transition pathways into leadership roles, supporting workforce re‐engagement following parental leave, and strengthening mentoring and networking opportunities. Leadership development should be accessible across career stages and employment arrangements, including for part‐time staff.

Equity in recruitment and promotion should be strengthened through transparent role advertising, diverse selection panels, and bias‐reduction strategies. These approaches can help build more inclusive workplace cultures and support sustainable leadership pipelines for women nurses and midwives.

For nursing managers and executive leaders, the findings highlight the need to move beyond individual‐focused leadership development and address the structural conditions that shape leadership opportunities. Embedding transparent career pathways, equitable recruitment practices, mentoring opportunities, flexible leadership models, and accountability mechanisms within workforce planning may strengthen leadership succession, workforce retention, and organizational performance.

#### 4.5.2. Implications for Research

Further research is needed to evaluate the effectiveness of system‐level and organizational‐level strategies for advancing nurses and midwives into leadership roles. Priority areas include leadership development programs, career pathways, nursing scope of practice, and workforce reforms designed to support leadership progression.

Research should also examine the outcomes of flexible leadership models, including part‐time, job‐sharing, fractional, and hybrid clinical leadership or clinical‐research roles, and their impact on workforce sustainability, career progression, retention, and patient outcomes.

Future research should investigate how gender intersects with ethnicity, cultural identity, migration status, and other social identities to shape leadership experiences in nursing and midwifery. Such work is needed to identify barriers and facilitators that may not be addressed through broad organizational equity initiatives alone and to inform more targeted approaches to leadership development and workforce inclusion.

## 5. Conclusions

Advancing women nurses and midwives into healthcare leadership requires a shift from individual‐focused approaches to evidence‐based systemic and organizational strategies. By addressing structural barriers and fostering empowerment, healthcare systems can build inclusive, resilient leadership pipelines that reflect the diversity and expertise of the nursing and midwifery workforce. This research will inform future directions of the national Advancing Women in Healthcare Leadership initiative, and Partnership Centre for Gender Equality in Leadership, funded by the National Health and Medical Research Council of Australia.

## Author Contributions

Ms Mihirika Surangi De Silva Pincha Baduge, Dr Belinda Garth, and Prof Helena J. Teede had full access to all of the data in the study and took responsibility for the integrity of the data and the accuracy of the data analysis.

Conceptualization: Mihirika Surangi De Silva Pincha Baduge, Belinda Garth, and Helena J. Teede.

Methodology: Belinda Garth and Helena J. Teede.

Formal analysis: Mihirika Surangi De Silva Pincha Baduge, Belinda Garth, Ifeoluwa Adesina, Kathryn Joseph, and Helena J. Teede.

Investigation: Belinda Garth, Ifeoluwa Adesina, and Kathryn Joseph.

Resources: Belinda Garth.

Writing–original draft: Mihirika Surangi De Silva Pincha Baduge, Belinda Garth, and Helena J. Teede.

Writing–review and editing: All authors.

Funding acquisition: Helena J. Teede and Leanne Boyd.

Project administration: Mihirika Surangi De Silva Pincha Baduge, Belinda Garth, and Helena J. Teede.

Supervision: Helena J. Teede, Belinda Garth, and Leanne Boyd.

## Funding

This project was supported by two National Health and Medical Research Council Partnership Grants (APP1191837 and APP2018718) and partner contributions.

Open access publishing was facilitated by Monash University, as part of the Wiley—Monash University agreement via the Council of Australasian University Librarians.

## Disclosure

In this study, the funding source was not involved in study design (data collection, analysis, and interpretation of data), in the writing of this report, or in the decision to submit the paper for publication.

## Conflicts of Interest

The authors declare no conflicts of interest.

## Data Availability

The data that support the findings of this study are available upon request from the corresponding author. The data are not publicly available due to privacy or ethical restrictions.
